# Internalizing and externalizing pathways to internet gaming disorder: the roles of anger and social anxiety

**DOI:** 10.3389/fpsyt.2026.1847115

**Published:** 2026-05-13

**Authors:** Mazen Omar Almulla, Ahmed Alduais, Abdullah Ahmed Almulla

**Affiliations:** 1 Department of Education and Psychology, King Faisal University, Al-Ahsa, Saudi Arabia; 2 Department of Psychology, Norwegian University of Science and Technology, Trondheim, Norway; 3 Department of Special Education, College of Education, King Faisal University, Al-Ahsa, Saudi Arabia

**Keywords:** anger, coping, internet gaming disorder, social anxiety, structural equational modelling

## Abstract

**Background:**

Internet Gaming Disorder (IGD) represents a significant behavioral health concern, yet the roles of internalizing and externalizing psychological vulnerabilities in its development remain underexplored, particularly in Arabic-speaking populations.

**Objective:**

This study examined anger and social anxiety as distinct externalizing and internalizing predictors of IGD severity in a Saudi Arabian community sample.

**Methods:**

A cross-sectional survey was administered to 303 participants (60.1% female; estimated mean age = 29.79 years, SD = 8.83) across five regions of Saudi Arabia. Participants completed the Internet Gaming Disorder Scale–Short Form (IGDS9-SF), a three-item Anger Screening Scale, and a two-item Social Anxiety screener. Hierarchical linear regression and structural equation modeling (SEM) were conducted to examine unique and incremental contributions of anger and social anxiety to IGD symptoms.

**Results:**

Anger and social anxiety were strongly intercorrelated (r = .86, p <.001) but demonstrated divergent patterns in multivariate models. Hierarchical regression indicated that both predictors contributed unique variance when entered simultaneously, with anger positively and social anxiety negatively predicting IGD after controlling for shared variance. However, SEM clarified that only social anxiety significantly predicted latent IGD severity (β = .32, p = .027), whereas anger did not (β = .07, p = .68). The final model explained approximately 13% of variance in IGD symptoms.

**Conclusions:**

Social anxiety was associated with IGD severity as a distinct internalizing correlate, consistent with avoidance-based coping and online social preference accounts. These preliminary, cross-sectional findings suggest that social anxiety warrants consideration in future IGD screening and research efforts in Arabic-speaking contexts.

## 1 Introduction

Problematic gaming is recognized in major diagnostic nosologies, although terminology and classification differ. The DSM-5-TR describes Internet Gaming Disorder (IGD) as a condition for further study, characterized by persistent and recurrent gaming associated with clinically significant impairment or distress, operationalized via nine proposed criteria with endorsement of five or more symptoms within a 12-month period (1). The ICD-11 includes IGD (code 6C51) as a disorder due to addictive behaviors, defined by impaired control over gaming, increasing priority given to gaming over other activities, and continuation or escalation despite negative consequences, with the behavior pattern typically evident for at least 12 months and producing significant functional impairment (2).

### 1.1 Internet gaming disorder as a behavioral health concern

Internet Gaming Disorder (IGD) is conceptualized as a behavioral addiction involving persistent and recurrent gaming that produces significant impairment in social, academic, occupational, or health domains (3–5). Symptom profiles include preoccupation with gaming, withdrawal, tolerance, unsuccessful attempts to reduce use, loss of interest in alternative activities, continued play despite harm, deception about gaming time, gaming to regulate negative affect, and marked functional impairment (3, 6–8). Epidemiological work suggests prevalence estimates of about 2–3% in the general population and substantially higher rates among adolescents and young adults, with elevated vulnerability reported in several Asian contexts (9–13). IGD correlates with depression, anxiety, cognitive difficulties, poor coping, social withdrawal, family stress, academic and occupational underperformance, sleep disturbance, physical inactivity, and reduced physical health (3, 6, 9, 14–16). Evidence from tertiary education populations further indicates that problematic and addictive internet-related behaviors may affect a notable proportion of university students, with overuse patterns being far more prevalent than clinically defined addiction (17).

Converging neurocognitive studies indicate that IGD shares key mechanisms with substance-related addictions, including altered reward processing, impaired executive control, and dysregulated emotion and motivation systems (3–5, 14, 18). Aberrant activity and connectivity in prefrontal, striatal, and limbic circuits accompany increased cue reactivity to gaming stimuli and heightened risk-taking (4, 7, 15). Psychosocial risk factors include impulsivity, emotional dysregulation, maladaptive coping, harsh or critical parenting, and adverse work or school environments, with robust comorbidity patterns involving depression, anxiety, and suicidal risk (9–13). Loneliness appears both a predictor and consequence of IGD, and has been linked to disrupted fronto-motor connectivity (6, 16).

### 1.2 Anger and IGD

A growing body of research links anger and related aggressive tendencies with IGD. Cross sectional work shows that higher IGDS9-SF scores co-occur with elevated state and trait anger, greater anger expression both outwardly and inwardly, and lower anger control, with anger expression in particular predicting IGD severity and supporting the view that gaming can function as a maladaptive strategy to manage negative affect (19, 20). Meta analytic evidence indicates a moderate association between IGD and aggression, especially in Asian samples and younger age groups, and longitudinal data suggest that increasing IGD symptoms prospectively predict subsequent increases in aggression, rather than the reverse, pointing to IGD as a potential risk factor for later aggressive behavior (21, 22). These links are embedded in broader psychosocial contexts: low social connectedness and loneliness partially mediate the relationships between IGD, anger, and aggression, while smartphone addiction and social appearance anxiety further intertwine with aggression and IGD risk (23–25). Emotion regulation research shows that people with IGD tend to rely on emotional suppression, report more anger, and are more prone to losing emotional control while gaming, particularly under provocation (26). It is important to note, however, that most prior research has operationalized anger in terms of trait anger or anger expression styles, whereas the present study employed a brief screener focused on functional impairment and risk of harm, which more closely reflects a clinical threshold of anger dyscontrol than the broader externalizing personality trait commonly referenced in the IGD literature (19, 20).

### 1.3 Social anxiety and IGD

A consistent body of evidence indicates that social anxiety is tightly linked with Internet Gaming Disorder (IGD) across adolescence and young adulthood, with socially anxious individuals more likely to engage in excessive or problematic gaming as they come to experience online environments as safer and less demanding than face to face interaction (27–30). Proposed mechanisms include a stronger preference for online social interactions, gaming motives centered on escape, coping and fantasy, and maladaptive metacognitions about gaming, as well as attentional biases toward game related social rewards and avoidance of real life social cues (27, 31). IGD shows high comorbidity with social anxiety disorder, depression, insomnia and related difficulties, and emerging findings suggest a mutually reinforcing relationship in which social anxiety increases vulnerability to IGD and, in turn, higher IGD severity is associated with worsening social anxiety and impaired offline relationships (28–30, 32). This association is further shaped by mediators and moderators such as fear of missing out, perceived social support and physical activity, indicating key targets for prevention and intervention efforts that address both social anxiety and problematic gaming within broader coping and support contexts (33–36).

### 1.4 The present study

The present study examined whether anger and social anxiety, conceptualized as indicative of externalizing and internalizing emotional profiles respectively, are differentially associated with IGD severity. Anger was operationalized as an externalizing affective indicator reflecting functional impairment and anger dyscontrol, whereas social anxiety was treated as an internalizing indicator capturing worry about social evaluation and its interference with daily functioning. Prior IGD research has examined internalizing and externalizing constructs largely in isolation, with few studies testing both domains simultaneously or in Arabic-speaking samples where culturally validated brief tools remain limited. The present study addressed this gap by examining associations among IGD symptoms, anger, and social anxiety in a Saudi community sample, and by testing whether social anxiety accounted for unique variance in IGD beyond anger when both were considered together. Given the cross-sectional design and the brief, unvalidated nature of the screening tools employed, all findings are interpreted as preliminary and hypothesis-generating rather than as evidence for established psychological pathways. The study offers exploratory evidence that may inform the design of more rigorously controlled future research in the region.

## 2 Methods

### 2.1 Participants

The study included 303 participants with no missing demographic data. The targeted minimum sample size was estimated using the Raosoft online sample size calculator with a 95% confidence level, 5% margin of error, and a conservative response distribution of 50%, which maximizes sample size requirements. Assuming a large national adult population, the recommended minimum sample size was 385 participants. The achieved sample of 303 participants corresponds to an estimated sampling error of approximately 5.6% at the same confidence level, which is considered acceptable for social and behavioral research surveys (37, 38). This sample size therefore provides adequate statistical power for correlational and regression analyses. For structural equation modeling with well-defined, simple factor structures, simulation studies suggest that samples of 100 to 200 can yield stable estimates when indicator loadings are moderate to high, and N = 300 is generally considered sufficient for models of low-to-moderate complexity (39). In the present model, the nine-indicator IGD factor demonstrated uniformly strong loadings, supporting estimation stability. The two- and three-item predictor scales showed weaker and less stable loadings, which is acknowledged as a limitation, particularly for the anger factor.

Of these, 182 were female (60.1%) and 121 were male (39.9%). Participants were distributed across regions of Saudi Arabia, including the Eastern Region (n = 181, 59.7%), Central Region (n = 42, 13.9%), Western Region (n = 35, 11.6%), Southern Region (n = 23, 7.6%), and Northern Region (n = 22, 7.3%). With respect to education, 163 participants (53.8%) held a bachelor’s degree, 38 (12.5%) a master’s degree, 10 (3.3%) a doctoral degree, and 92 (30.4%) reported a high school qualification or below. Regarding marital status, 150 (49.5%) were single, 123 (40.6%) married, 22 (7.3%) divorced, and 8 (2.6%) widowed. Employment status showed that 127 participants (41.9%) were employed, 23 (7.6%) self-employed, 89 (29.4%) students, and 64 (21.1%) unemployed (see **Table 1**).

**Table 1:** Sample characteristics of study participants. (N = 303).

Variable	Category	n	%
Sex	Female	182	60.1
	Male	121	39.9
Age Category	Adolescents (<18 years)	17	5.6
	Emerging adults (18–24 years)	82	27.1
	Young adults (25–30 years)	69	22.8
	Adults (31–40 years)	89	29.4
	Middle-aged adults (>40 years)	46	15.2
Region	Central	42	13.9
	Eastern	181	59.7
	Northern	22	7.3
	Southern	23	7.6
	Western	35	11.6
Educational Level	High school or below	92	30.4
	Bachelor’s degree	163	53.8
	Master’s degree	38	12.5
	Doctorate	10	3.3
Marital Status	Single	150	49.5
	Married	123	40.6
	Divorced	22	7.3
	Widowed	8	2.6
Employment Status	Employee	127	41.9
	Self-employed	23	7.6
	Student	89	29.4
	Unemployed	64	21.1

Estimated mean age = 29.79 years (SD = 8.83), derived from midpoint-based grouped data formulas.

Participants represented a broad developmental range, including adolescents (n = 17, 5.6%), emerging adults (n = 82, 27.1%), young adults (n = 69, 22.8%), adults (n = 89, 29.4%), and middle-aged adults (n = 46, 15.2%). Because age was collected in categories, an estimated mean age and standard deviation were calculated using midpoint-based grouped data formulas. The estimated mean age was 29.79 years, with an estimated standard deviation of 8.83 years, providing a reasonable summary of the age distribution in the absence of raw continuous age data (see **Table 1**).

Inclusion criteria required participants to be residing in Saudi Arabia, able to understand and complete the survey, and willing to provide informed consent. The study targeted a non-clinical community sample; therefore, participants who reported a current or previously diagnosed psychiatric or neurological disorder were not eligible to participate. This criterion was based on self-report and no clinical verification was conducted.

### 2.2 Measures

#### 2.2.1 Internet gaming disorder scale–short form

The IGDS9-SF (40) comprises nine items reflecting the DSM-5 criteria for Internet Gaming Disorder and assesses gaming behavior over the past twelve months. Items are rated on a five-point Likert scale from 1 (Never) to 5 (Very Often), producing a total score ranging from 9 to 45, with higher scores indicating greater severity of gaming-related problems. The instrument has demonstrated strong psychometric performance, including a stable one-factor structure and high internal consistency (α approximately.87 to.89 across independent samples). Although primarily designed to capture severity rather than provide a clinical diagnosis, prior work suggests that endorsement of at least five items at the highest response frequency may indicate probable disorder status. The scale has been widely used in both community and research contexts and is supported by extensive validation evidence., including an Arabic validation study conducted with a Saudi community sample (41). In the present sample, the scale demonstrated excellent internal consistency (Cronbach’s α = .89).

#### 2.2.2 Anger screening scale

The anger screening measure was obtained from eMentalHealth.ca, a public mental health resource providing community screening tools. The instrument includes three dichotomous items (Yes = 1, No = 0) assessing core anger experiences and functional impairment (e.g., frequency of anger, interference with daily functioning, and anger associated with risk of harming others). The items follow the DSM-IV clinical convention of identifying symptom presence and determining whether symptoms cause significant problems in daily life. The developers explicitly state that the screener has not been formally validated and no normative data or psychometric indices are available; it is intended for preliminary screening and psychoeducation rather than diagnostic determination (42). The present study employed this screener alongside the IGDS9-SF as part of a psychometric evaluation of brief Arabic screening tools (41). In the present sample, internal consistency was low (Cronbach’s α = .23), consistent with expectations for brief, heterogeneous screening tools.

#### 2.2.3 Social anxiety

The Social Anxiety screener was obtained from eMentalHealth.ca, which provides freely accessible community mental health screening tools intended for public education and early identification of potential concerns (43). The measure consists of two Yes/No items assessing worry about embarrassment or humiliation in social situations and whether this worry interferes with daily functioning. Items are scored 0 or 1 and summed to produce a total ranging from 0 to 2, with higher scores reflecting greater endorsement of social anxiety features. Consistent with the developers’ guidance, the screener is not a diagnostic instrument and no formal validation or population norms are available (eMentalHealth.ca, 2025). In the present sample, internal consistency was modest (Cronbach’s α = .40), consistent with the two-item screening format.

### 2.3 Procedure and ethics

Data were collected over a three-month period between August and October 2025 using an online survey hosted on Google Forms. All study instruments were administered in Modern Standard Arabic, and the survey link was distributed through professional and academic networks, including school and university staff groups across multiple regions of Saudi Arabia. Prior to data collection, the survey was reviewed for clarity, piloted with a small group to confirm comprehension and technical functionality, and then finalized for administration. Informed consent was obtained electronically. The opening page of the survey explained the study purpose, confidentiality procedures, voluntary participation, and the right to withdraw at any stage, and participants were required to indicate consent before proceeding. For adolescent participants, parental consent was additionally obtained in accordance with ethical guidance. Ethical approval was granted by the Institutional Review Board at King Faisal University, Saudi Arabia (approval number: KFU-2025-ETHICS3520).

### 2.4 Statistical analysis plan

All analyses were conducted in jamovi version 2.3 (44), which runs on R version 4.1 (45). For all study variables, we computed means, standard deviations, skewness, and kurtosis using the Exploration - Descriptives module in jamovi. These analyses provided an initial check of distributional assumptions for subsequent parametric procedures.

Internal consistency for the IGDS9-SF and the Anger Screening Scale was evaluated using Cronbach’s alpha and McDonald’s omega, implemented through jamovi’s Reliability module and the psych package in R (46). For the two-item Social Anxiety screener, reliability was assessed using the inter item Pearson correlation and the Spearman Brown coefficient, following standard recommendations for very brief scales. Agreement-oriented indices and kappa-related considerations were informed by the irr package (47), work on agreement metrics in clinical applications (48), and supporting tools such as ClinicoPath (49) and kappaSize (50, 50).

Bivariate associations among IGD, anger, and social anxiety were estimated using Pearson correlation coefficients with the Regression - Correlation Matrix module in jamovi. Confidence intervals and significance tests were produced within jamovi, with conceptual and computational support from psych (46) and car for regression-related utilities where needed (51).

To examine the unique and incremental contributions of anger and social anxiety to IGD severity, we fitted hierarchical linear regression models with the IGDS9-SF total score as the dependent variable using the Regression - Linear Regression module in jamovi. In Step 1, demographic covariates (sex, age category, and region) were entered where theoretically justified. In Step 2, Anger Screening Scale scores were entered. In Step 3, Social Anxiety scores were added to test incremental prediction beyond anger. Model fit statistics, standardized regression coefficients, and 95 percent confidence intervals were reported. Multicollinearity and basic regression assumptions were checked using standard outputs and diagnostics informed by the car package (51).

Finally, we estimated a latent-variable structural equation model specifying IGD as a latent outcome predicted by latent anger and social anxiety factors. This model was fitted in jamovi using the SEMLj module (52), which interfaces with the lavaan package in R (53). Global fit indices (χ², CFI, TLI, RMSEA, SRMR) and standardized factor loadings were obtained via SEMLj and lavaan, with auxiliary functions from semTools for reliability and additional fit statistics (54). Path diagrams for presentation were generated using semPlot (55).

## 3 Results

### 3.1 Descriptive statistics

Across the full sample of 303 (M_age_ = 29.79, SD_age_ = 8.83) participants with no missing data, descriptive statistics indicated that the Anger Scale total demonstrated a mean of 1.67 (SD = 0.79), reflecting generally low to moderate endorsement of anger-related experiences, where higher scores indicate greater negative influence of anger. The IGDS9-SF total mean was 17.62 (SD = 7.56) within the possible range of 9 to 45, suggesting relatively low average levels of Internet Gaming Disorder symptoms in this sample; however, scores varied widely, and meaningful risk is typically expected when at least five of the nine criteria are endorsed at the “Very Often” level. The Social Anxiety total mean was 1.47 (SD = 0.64), indicating generally lower endorsement of symptoms, with higher scores representing greater symptom likelihood. Precision estimates showed narrow standard errors and stable confidence intervals for each measure (Anger SE = 0.05, 95% CI [1.58, 1.76]; IGDS9-SF SE = 0.43, 95% CI [16.77, 18.48]; Social Anxiety SE = 0.04, 95% CI [1.40, 1.54]). Distribution indices indicated that Anger Total scores were approximately symmetric (skewness = −0.10; kurtosis = −0.44), IGDS9-SF scores were positively skewed with near-normal kurtosis (skewness = 0.79; kurtosis = 0.21), and Social Anxiety scores showed mild negative skew with slightly flatter distribution (skewness = −0.80; kurtosis = −0.40). Shapiro–Wilk tests were statistically significant for all total scales (Anger W = 0.86, p <.001; IGDS9-SF W = 0.92, p <.001; Social Anxiety W = 0.73, p <.001), indicating departures from normality; however, these deviations are common for symptom measures and, given the sufficiently large sample size, do not contraindicate the use of parametric analyses. See **Table 2** for detailed statistics.

**Table 2:** Descriptive statistics and normality tests for study variables.

Variable	M	SE	95% CI (LL, UL)	SD	Skewness	Kurtosis	W	P
Anger Item 1	0.83	0.02	0.79, 0.88	0.37	−1.81	1.30	0.45	<.001
Anger Item 2	0.63	0.03	0.58, 0.69	0.48	−0.56	−1.70	0.61	<.001
Anger Total	1.67	0.05	1.58, 1.76	0.79	−0.10	−0.44	0.86	<.001
IGDS9-SF Item 1	2.17	0.06	2.04, 2.30	1.13	0.60	−0.56	0.85	<.001
IGDS9-SF Item 2	1.84	0.06	1.72, 1.97	1.08	1.24	0.95	0.76	<.001
IGDS9-SF Item 3	2.08	0.07	1.95, 2.21	1.15	0.98	0.20	0.82	<.001
IGDS9-SF Item 4	1.93	0.06	1.81, 2.06	1.12	1.02	0.15	0.79	<.001
IGDS9-SF Item 5	1.98	0.07	1.85, 2.11	1.16	1.02	0.13	0.80	<.001
IGDS9-SF Item 6	1.86	0.06	1.74, 1.98	1.08	1.23	0.84	0.77	<.001
IGDS9-SF Item 7	1.78	0.07	1.65, 1.91	1.15	1.43	1.02	0.71	<.001
IGDS9-SF Item 8	2.31	0.07	2.16, 2.46	1.29	0.62	−0.72	0.85	<.001
IGDS9-SF Item 9	1.67	0.07	1.54, 1.80	1.14	1.62	1.51	0.64	<.001
IGDS9-SF Total	17.62	0.43	16.77, 18.48	7.56	0.79	0.21	0.92	<.001
Social Anxiety Item 1	0.61	0.03	0.56, 0.67	0.49	−0.47	−1.79	0.62	<.001
Social Anxiety Item 2	0.36	0.03	0.30, 0.41	0.48	0.60	−1.65	0.61	<.001
Social Anxiety Total	1.47	0.04	1.40, 1.54	0.64	−0.80	−0.40	0.73	<.001

CI, confidence interval; LL, lower limit; UL, upper limit. The CI of the mean assumes sample means follow a *t* distribution with *N − 1* degrees of freedom. Exact participant ages were not available; therefore, age was estimated using standard grouped-data procedures. Numerical midpoints were assigned to each age category (<18 = 15; 18–24 = 21; 24–30 = 27; 30–40 = 35; ≥40 = 45), and the estimated mean age was calculated using.


$$\bar{x}=\frac{\sum f_{i}m_{i}}{\sum f_{i}}$$

where $f_{i}$ represents the frequency of each age group and $m_{i}$ its midpoint. The estimated standard deviation was computed using


$$SD=\sqrt{\frac{\sum f_{i}{\left(m_{i}-\bar{x}\right)}^{2}}{\sum f_{i}}}$$

### 3.2 Categories of internet gaming disorder, anger, and social anxiety

For interpretive clarity and to support meaningful comparison across severity levels, IGDS9-SF scores were classified using an operational severity framework informed by prior clinical guidance on gaming disorder assessment (**Table 3**). Classification was based on response endorsement patterns rather than raw summed scores. Participants were categorized as disordered gamers when five or more items were endorsed at the “very often” level, reflecting consistent and pervasive symptom presence. Severe symptoms were defined by endorsement of three to four “very often” responses, whereas elevated symptoms were indicated by endorsement of one to two “very often” responses or at least three “often” responses, representing notable but less pervasive difficulties. Moderate symptoms were defined by endorsement of one to two “often” responses or three or more “sometimes” responses, indicating emerging risk. Mild symptoms reflected predominantly “never” or “rarely” responses, with none of the higher threshold criteria triggered.

**Table 3:** Categorical distribution of study measures (N = 303).

Measure/category	n	%
IGDS9-SF severity		
Mild	152	50.17
Moderate	81	26.73
Elevated	54	17.82
Severe	11	3.63
Disordered	5	1.65
Anger		
Anger may be causing problems	180	59.41
May have problems with anger	104	34.32
Not having problems with anger	19	6.27
Social anxiety		
Likely social anxiety symptoms	166	54.79
Possible social anxiety symptoms	113	37.29
Unlikely social anxiety symptoms	24	7.92

For the Anger Scale, participants were categorized according to the number of “Yes” responses. Those endorsing two or three items were classified as “Anger may be causing problems,” while endorsement of a single item was categorized as “May have problems with anger.” Participants who responded “No” to all three items were classified as “Not having problems with anger.” These classifications reflect symptom endorsement and perceived impairment rather than clinical diagnosis.

Finally, the two-item social anxiety measure was categorized for descriptive and comparative purposes. Endorsement of both items indicated likely social anxiety symptoms, endorsement of one item indicated possible symptoms, and endorsement of neither item suggested unlikely symptoms. Across all three measures, these categories are intended to describe symptom severity patterns only and do not constitute diagnostic classification.

### 3.3 Reliability

Internal consistency analyses indicated excellent reliability for the IGDS9-SF. The mean scale score was 1.96 (SD = 0.84), with Cronbach’s alpha of.89 and McDonald’s omega of.90, supporting strong coherence among the nine items. The Anger Scale showed a mean of 0.56 (SD = 0.26) and demonstrated weak internal consistency (α = .23, ω = .25), which is expected for very brief and heterogeneous screening tools and is acceptable for its purpose as a short-risk indicator rather than a full clinical scale. The two-item Social Anxiety measure had a mean of 0.49 (SD = 0.38) and produced α = .40 and ω = .40, values consistent with short two-item screening formats. Because short dichotomous symptom checklists require agreement-based reliability assessment rather than traditional alpha alone, inter-item and agreement statistics were also examined. The two items showed statistically significant but modest association (r = .25, 95% CI [.14,.35], p <.001; ρ = .25, p <.001), with 58 percent raw agreement and Cohen’s κ = .22 (z = 4.36, p <.001), reflecting meaningful but not redundant overlap consistent with a brief screening indicator rather than a diagnostic instrument. See **Table 4** for detailed statistics.

**Table 4:** Internal consistency and interrater reliability of study measures.

Scale	α	ω	Inter-item pearson r	Inter-item spearman ρ	Cohen’s κ	Agreement %
Anger	.23	.25	—	—	—	—
IGDS9-SF	.89	.90	—	—	—	—
Social Anxiety	.40	.40	.25***	.25***	.22***	58

α = Cronbach’s alpha; ω = McDonald’s omega. For the two-item Social Anxiety indicator, reliability was evaluated using inter-item association and Cohen’s kappa instead of alpha, which is not appropriate for two-item measures. Agreement percentage reflects raw concordance between items. ***p <.001.

### 3.4 Correlations

Zero-order Pearson correlations were conducted to examine the associations among anger, social anxiety, and Internet Gaming Disorder symptoms. Anger was not significantly correlated with IGDS9-SF total scores (r = .02, p = .67), indicating no meaningful bivariate association between anger and IGD symptoms. Similarly, social anxiety was not significantly correlated with IGDS9-SF total scores (r = −.08, p = .18). However, anger and social anxiety were very strongly and positively correlated (r = .86, p <.001), indicating substantial overlap between these two constructs at the total-score level. These findings suggest that although anger and social anxiety are highly related to each other, neither demonstrated a significant zero-order association with IGD symptoms prior to multivariate modeling. See **Table 5** for detailed statistics.

**Table 5:** Zero-order pearson correlations among study variables (N = 303).

Variable	1	2	3
1. Anger Total	—		
2. IGDS9-SF Total	.02 (p = .67)	—	
3. Social Anxiety Total	.86 (p <.001)	−.08 (p = .18)	—

Values represent Pearson correlation coefficients with corresponding *p* values in parentheses. Higher scores on all measures indicate greater symptom endorsement.

### 3.5 Regression analyses

A hierarchical linear regression analysis was conducted to examine the predictive contributions of anger and social anxiety to Internet Gaming Disorder symptoms, with demographic variables subsequently added to evaluate model stability. Model 1 included sex only and did not significantly predict IGDS9-SF scores (R² = .001, p = .59). In Model 2, anger was added, but the model remained non-significant and accounted for minimal variance (R² = .002, p = .61). In Model 3, social anxiety was introduced. This resulted in a statistically significant model improvement (ΔR² = .038, F(1, 299) = 11.69, p <.001), and both predictors became significant: higher anger was associated with higher IGD symptoms (β = .36, p = .002), whereas higher social anxiety was associated with lower IGD symptoms when anger was controlled (β = −.39, p <.001).

Subsequent models sequentially added demographic variables to test the robustness of these effects. Adding marital status (Model 4) did not materially change the predictive pattern. When age group was added (Model 5), explained variance increased meaningfully (R² = .101, p <.001), with older age groups reporting lower IGD compared to adolescents; however, anger remained a significant positive predictor and social anxiety remained a significant negative predictor. Education (Model 6) and employment (Model 7) did not contribute additional unique variance, and their inclusion did not alter the strength or direction of the anger or social anxiety effects. The final fully adjusted model explained approximately eleven percent of variance in IGD symptoms (R² = .114), with anger remaining a significant positive predictor (β ≈.29, p ≈.01) and social anxiety a significant negative predictor (β ≈ −.27, p ≈.02). This pattern indicates that, despite strong overlap between the two psychological variables, each contributed unique variance in opposing directions once entered together in the model. See **Table 6** for detailed statistics.

**Table 6:** Hierarchical linear regression predicting internet gaming disorder (IGDS9-SF total; N = 303).

Model	Predictors added	R²	ΔR²	F for ΔR²	p	Key findings
1	Sex	.001	—	0.29	.59	Sex not significant
2	+ Anger	.002	.001	0.26	.61	Anger still not significant
3	+ Social Anxiety	.039	.038	11.69	<.001	Anger (+) and Social Anxiety (−) both significant
4	+ Marital Status	.045	.006	1.91	.17	Pattern unchanged
5	+ Age Group	.101	.056	4.55	.001	Age significant; Anger (+) & Social Anxiety (−) remain significant
6	+ Education	.114	.013	1.44	.23	Pattern unchanged
7	+ Employment	.114	.000	0.01	.93	Final model stable

Standardized effects in models where both predictors entered: Model 3: Anger β = .36, p = .002; Social Anxiety β = −.39, p <.001; Model 5: Anger β ≈.29, p ≈.01; Social Anxiety β ≈ −.28, p ≈.02; and Model 7 (final): Anger β = .29, p = .010; Social Anxiety β = −.27, p = .020. Dependent variable is IGDS9-SF total score. R² is cumulative explained variance. ΔR² reflects variance gained by each block. All models used the same dependent variable; predictors were entered sequentially based on theoretical relevance.

### 3.6 Structural equation modelling of IGD with anger and social anxiety

**Figure 1:**
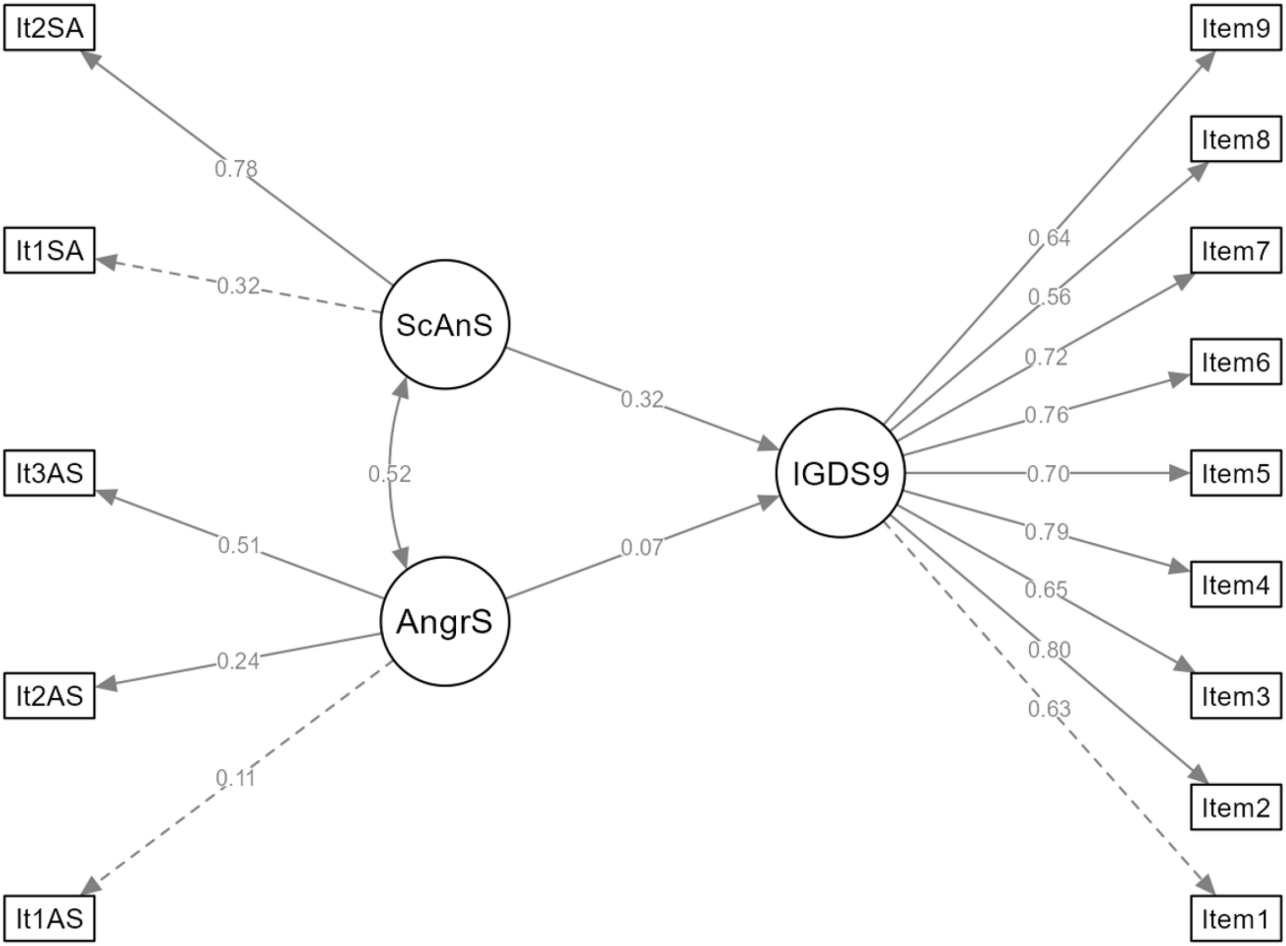
Structural equation model examining anger and social anxiety as predictors of internet gaming disorder. IGDS9, Internet Gaming Disorder Scale 9-Item Short Form latent factor; ScAnS, Social Anxiety latent factor; AngrS, Anger latent factor; It1SA and It2SA, Social Anxiety observed indicators; It1AS, It2AS, and It3AS, Anger observed indicators; Item1 to Item9, IGDS9-SF observed indicators. Solid paths represent statistically significant standardized factor loadings and structural paths, while dashed paths indicate nonsignificant estimates.

A latent path model was specified in which Anger Scale (three items: Item1AS–Item3AS) and Social Anxiety Scale (two items: Item1SA–Item2SA) predicted a latent IGDS9−SF factor (nine items: Item1–Item9) (**Figure 1**). The model was estimated with maximum likelihood (ML) using NLMINB optimization on 303 participants, with 45 free parameters. Standard errors were set to “Standard.” The model converged successfully after 97 iterations. Model fit was acceptable: χ²(74) = 237.24, p <.001, SRMR = .057, RMSEA = .085 with 90% CI [.073,.098], and RMSEA close-fit test p = 1.85 × 10^-6^. Comparative indices were CFI = .88, TLI/NNFI = .86, NFI = .84, RFI = .80 and IFI = .88, with PNFI = .68. The GFI was.96 and AGFI was.94, with PGFI = .60 and McDonald’s MFI = .76. Information criteria indicated a reasonably parsimonious model (AIC = 9114.03, BIC = 9281.15, SABIC = 9138.44). Hoelter’s critical N values were 122.43 (α = .05) and 135.36 (α = .01). The latent outcome IGDS9−SF had R² = .13, indicating that Anger Scale and Social Anxiety Scale together explained about 13% of its variance.

Consistent with the hierarchical regressions, Social Anxiety Scale showed a significant positive effect on IGDS9−SF (unstandardized b = 1.44, SE = 0.65, 95% CI [0.16, 2.72], β = .32, z = 2.20, p = .027). Anger Scale was not a significant predictor at the latent level (b = 1.23, SE = 2.97, 95% CI [−4.60, 7.06], β = .07, z = 0.41, p = .68). Thus, only social anxiety uniquely predicted latent IGD severity in this model. All IGDS9−SF indicators loaded significantly and strongly on the latent IGD factor. Standardized loadings were.63 for Item1,.80 for Item2,.65 for Item3,.79 for Item4,.70 for Item5,.76 for Item6,.72 for Item7,.56 for Item8 and.64 for Item9 (all z ≥ 8.63, all p <.001). This indicates that the nine items capture a common IGD construct with good representation. For Social Anxiety Scale, Item1SA and Item2SA showed moderate to high loadings (β = .32 and.78 respectively). Item2SA was statistically significant (b = 2.39, SE = 1.01, 95% CI [0.42, 4.36], z = 2.38, p = .017), while Item1SA functioned as the reference indicator (loading fixed to 1.00). For Anger Scale, Item1AS was the reference indicator (β = .11). Item2AS and Item3AS had modest loadings (β = .24 and.51, respectively) that were not statistically significant at the.05 level (z ≈ 1.12, p ≈.26), indicating weaker measurement precision for the anger factor.

Residual variances for IGDS9−SF items were moderate, with standardized values from.37 to.68, indicating that between one third and two thirds of each item’s variance was unique rather than shared with the latent factor. The latent variance of IGDS9−SF was significant (var = 0.44, SE = 0.08, 95% CI [0.29, 0.59], β = .87, z = 5.60, p <.001). Variances for Anger Scale and Social Anxiety Scale were small and not clearly different from zero at the.05 level, which is consistent with the small scale ranges and limited reliability for these short scales. The covariance between Anger Scale and Social Anxiety Scale was positive but non-significant (cov = 0.0035, SE = 0.0033, 95% CI [−0.0031, 0.0100], β = .52, z = 1.04, p = .30). Model-implied latent covariances showed that IGDS9SF was moderately associated with both Anger Scale (cov = 0.24) and Social Anxiety Scale (cov = 0.36), in line with the observed correlations reported earlier. All observed item intercepts were positive and statistically significant (z ≥ 8.83, all p <.001). For example, IGDS9SF items had intercepts ranging from 1.67 (Item9) to 2.31 (Item8), and social anxiety items from 0.36 to 0.61. This pattern indicates non-zero baseline endorsement of symptoms even at the latent means fixed to zero.

Reliability indices supported good internal consistency for IGDS9−SF (α = .89, ω ≈.89, AVE = .48). Social Anxiety Scale showed modest reliability (α = .40, ω ≈.48, AVE = .35), whereas Anger Scale reliability was low (α = .23, ω ≈.22, AVE = .11), which should be considered when interpreting its non-significant structural path. HTMT ratios suggested that the three constructs were related yet distinct: Anger Scale – Social Anxiety Scale = .68, Anger Scale –IGDS9SF = .40 and Social Anxiety Scale– IGDS9−SF = .40, all below common discriminant validity thresholds. Mardia’s skewness (coefficient = 36.24, χ² = 1829.9, df = 560, p < 1 × 10^-134^) and kurtosis (coefficient = 271.70, z = 19.62, p < 1 × 10^-85^) indicated substantial deviations from multivariate normality, justifying the use of robust checks in earlier analyses and supporting cautious interpretation of ML-based indices.

Modification indices suggested that allowing residual covariances among several IGD items and between one anger item and IGD items could improve fit. The largest indices involved correlated errors between Item7 and Item9 (MI = 44.24, sEPC(all) = 0.43), Item3 and Item9 (MI = 20.19), Item4 and Item8 (MI = 19.00), and Item5 and Item7 (MI = 15.11). Additional suggested correlations included Item3 with Item8, Item1 with Item8, Item1AS with Item2, and Item2 with Item5 (all MI ≥ 10.83). These patterns may reflect specific shared content among IGD items, but were not incorporated into the final model in order to retain a theory-driven, parsimonious specification.

In summary, the SEM indicates that social anxiety has a significant and meaningful positive association with latent IGD severity (β = .32, p = .027), whereas anger does not have a significant direct effect at the latent level (β = .07, p = .68). IGDS9SF is well represented by its nine indicators and shows strong reliability, while the short anger scale is measured less precisely. The final structural relations and standardized factor loadings are illustrated in the path diagram (**Figure 1**), where statistically significant paths are shown by solid arrows and non-significant paths by dashed arrows.

## 4 Discussion

### 4.1 Summary of findings

The present study examined whether anger and social anxiety, as indicators of externalizing and internalizing emotional profiles, were differentially associated with IGD severity in a Saudi Arabian community sample. Results indicated that while anger and social anxiety were strongly intercorrelated at the bivariate level (r = .86), they demonstrated divergent patterns in multivariate models. Hierarchical regression analyses showed that, when entered simultaneously, anger and social anxiety contributed unique variance to IGD symptoms in opposing directions, a pattern attributable to suppression effects given the very high intercorrelation between predictors. Structural equation modeling indicated that only social anxiety was significantly associated with latent IGD severity (β = .32, p = .027), whereas anger was not (β = .07, p = .68). Given the cross-sectional design and the limited psychometric properties of the screening tools employed, these findings should be interpreted as preliminary and hypothesis-generating. They tentatively suggest that social anxiety may be a more proximal internalizing correlate of IGD than anger dyscontrol, a possibility that warrants investigation with validated measures and longitudinal designs. The study also offers exploratory evidence for the utility of a brief Arabic social anxiety indicator in IGD research.

### 4.2 Interpretation within theory

The observed association between social anxiety and IGD aligns with theoretical frameworks emphasizing avoidance-based coping and preference for online social interaction as key mechanisms linking internalizing vulnerabilities to problematic gaming behaviors (27). According to the social compensation hypothesis, individuals with elevated social anxiety may gravitate toward online gaming environments because these contexts offer reduced social evaluative threat, greater perceived control over self-presentation, and opportunities for achievement and social connection without the demands of face-to-face interaction (56, 57). Gaming may thus function as a maladaptive avoidance strategy that temporarily alleviates social distress while simultaneously reinforcing withdrawal from real-world social contexts and perpetuating a cycle of increasing isolation and gaming engagement (58). This compensatory use pattern has been consistently documented across cultural contexts, with socially anxious individuals reporting stronger preferences for online communication, greater reliance on escape and coping motives, and heightened vulnerability to IGD symptomatology (27, 31). The present findings extend this literature by showing that social anxiety is associated with IGD severity even when co-occurring externalizing indicators are statistically controlled, consistent with the distinctiveness of internalizing correlates in IGD research, though the cross-sectional design precludes causal interpretation.

The weaker role of anger in the latent structural model contrasts with prior research linking anger expression, aggression, and IGD symptoms in Asian samples (22, 59). This discrepancy may reflect methodological differences, including the use of a brief, unvalidated anger screener with limited psychometric properties (α = .23) that may not have adequately captured trait anger or anger dysregulation constructs assessed in prior studies. From a theoretical perspective, anger and externalizing behaviors are understood within the general model of addiction as downstream consequences of impaired self-regulation, reward sensitivity, and executive dysfunction (4, 60). The interaction-of-person-affect-cognition-execution (I-PACE) model posits that predisposing vulnerabilities—including both internalizing conditions such as social anxiety and externalizing traits such as impulsivity—interact with gaming-specific cognitions and affective responses to drive compulsive use patterns (61). The divergence between the present findings and I-PACE predictions for externalizing pathways is partly attributable to this measurement distinction: the I-PACE model references dispositional trait anger and impulsivity, whereas the screener used here assessed functional impairment and anger dyscontrol at a clinical threshold, capturing a narrower expression of the externalizing domain. Although the present study did not find a robust direct effect of anger on IGD at the latent level, anger may still operate indirectly through its association with emotion regulation difficulties, stress reactivity, and co-occurring internalizing symptoms. Future research employing comprehensive anger assessments and testing mediation pathways is needed to clarify the role of externalizing vulnerabilities in IGD development within Arabic-speaking populations.

The divergence in the directionality of results between the hierarchical regression and the SEM warrants explicit attention. In the regression model, social anxiety emerged as a negative predictor of IGD when anger was simultaneously controlled, whereas in the SEM social anxiety was a significant positive predictor of latent IGD severity. This apparent sign reversal is a classical statistical suppression effect arising from the very high intercorrelation between predictors (r = .86): when two highly correlated predictors are entered together in ordinary least squares regression, each absorbs shared variance from the other, which can reverse or attenuate standardized coefficients relative to their true-score relationships (62). The SEM framework, by contrast, models each construct as a latent variable estimated from its indicators, thereby partitioning true-score variance from measurement error and yielding path coefficients that are less susceptible to this artefact. Accordingly, the latent-variable SEM results are considered the more theoretically interpretable representation of the internalizing pathway, with social anxiety showing a genuine positive association with IGD severity once measurement error is separated from construct-level variance. The regression findings, while informative about unique predictive contributions under collinearity, should be interpreted cautiously given the suppression context.

The low internal consistency of the Anger Screening Scale (α = .23) also has direct implications for the SEM results. Low alpha implies high measurement error relative to true-score variance, which attenuates the latent factor variance and inflates the standard errors of structural paths emanating from that factor. This is consistent with the non-significant and imprecise anger path observed in the present SEM (β = .07, SE = 2.97, p = .68). The anger scale items tap qualitatively distinct aspects of anger experience (frequency, daily interference, risk of harm), which may not share sufficient common variance to define a reliable latent factor. In contrast, the Social Anxiety screener, despite modest alpha (α = .40), produced a significant structural path (β = .32, p = .027), partly because Item 2 carried strong indicator variance (β = .78). Future research should treat these brief screeners as observed composite risk indicators rather than fully specified latent factors, and should employ validated, multi-item measures of trait anger and social anxiety to permit more reliable latent variable modeling.

### 4.3 Implications for research

These findings highlight the value of incorporating brief, accessible social anxiety indicators in large-scale IGD research, particularly in contexts where comprehensive diagnostic assessments may be impractical. The two-item social anxiety screener demonstrated acceptable performance as a risk indicator despite modest internal consistency, suggesting that even brief measures can contribute meaningfully to multivariate risk models when targeting well-defined symptom domains. More broadly, the study underscores the importance of considering both internalizing and externalizing pathways in IGD research, as exclusive focus on either domain may obscure unique risk mechanisms. The significant positive association between social anxiety and IGD, even when controlling for anger, supports the adoption of multivariate frameworks that integrate affective, cognitive, and behavioral predictors across traditional diagnostic boundaries (58, 60). This approach is particularly relevant for advancing IGD research in Arabic-speaking contexts, where validated Arabic-language assessment tools are increasingly available (41, 63, 64) but comprehensive studies examining multiple psychological risk factors remain limited. Future research should prioritize culturally adapted measures assessing emotion regulation, coping motives, and interpersonal functioning to enable more nuanced investigation of IGD pathways in the region.

### 4.4 Practical implications

Although the cross-sectional design precludes causal conclusions, the observed association between social anxiety and IGD is consistent with theoretical accounts that position social anxiety as a correlate of problematic gaming, and raises the possibility that social anxiety screening may be a relevant component of broader IGD risk assessment efforts (27, 28). Should longitudinal research confirm a prospective association, prevention and early intervention programs might consider addressing social anxiety alongside gaming behavior, for example through approaches that build offline social competence, reduce avoidance behaviors, and develop adaptive coping skills for managing social distress (33, 35). Given the elevated prevalence of IGD among young people in Saudi Arabia and neighboring Arab states—with rates ranging from 10% to 45% depending on assessment methods and populations (65–67)—these preliminary findings may inform the design of future hypothesis-driven intervention research in the region.

### 4.5 Limitations

Several limitations warrant consideration when interpreting these findings. First, the cross-sectional design precludes causal inference, and the directionality of associations among anger, social anxiety, and IGD cannot be determined from the present data. Longitudinal research is needed to clarify whether social anxiety prospectively predicts IGD onset and escalation, or whether problematic gaming exacerbates social withdrawal and anxiety over time, as suggested by reciprocal models (28, 29). Second, both the Anger Screening Scale and the Social Anxiety screener were obtained from a public mental health website and have not undergone formal psychometric validation. Neither instrument has published normative data, established factor structure, or documented criterion validity. Using such tools as primary indicators in a latent variable SEM is a substantive limitation: the anger screener’s very low internal consistency (α = .23) means that the latent anger factor is largely defined by measurement error rather than construct-relevant variance, rendering its structural path coefficients unreliable. The social anxiety screener’s modest consistency (α = .40) similarly constrains the precision of its latent factor. These limitations affect the interpretability of the SEM results and preclude strong conclusions about the relative magnitudes of the externalizing and internalizing pathways. Future studies should employ validated, multi-item measures of trait anger (e.g., the State-Trait Anger Expression Inventory) and social anxiety (e.g., the Social Interaction Anxiety Scale) to permit more reliable latent variable modeling and stronger inferences about differential pathway effects. Third, while the sample was diverse in age and regional representation, participants were drawn exclusively from Saudi Arabia, and the exclusion of individuals with diagnosed psychiatric or neurological disorders may have restricted variability in key constructs and limited generalizability to clinical populations. Additionally, reliance on self-report measures introduces potential biases related to social desirability, recall accuracy, and subjective interpretation of symptoms. The estimated mean age derived from categorical data, though methodologically appropriate, also represents an approximation rather than precise measurement.

### 4.6 Future directions

Building on these findings, future research should pursue several complementary directions to advance understanding of IGD etiology in Arabic-speaking populations. First, planned analyses will examine gaming motives (e.g., escape, achievement, social connection) and self-compassion as additional psychological mechanisms that may mediate or moderate the relationships between social anxiety, anger, and IGD. Investigating how specific gaming motivations interact with affective vulnerabilities will provide a more nuanced account of individual pathways to problematic use. Second, longitudinal designs are essential to establish temporal ordering and test prospective prediction of IGD symptom trajectories from baseline psychological risk factors. Such designs would also enable examination of bidirectional effects and identification of critical developmental windows for intervention. Third, extension to clinical samples with diagnosed IGD, as well as comparative studies across Arab states, would enhance understanding of cultural and contextual influences on IGD risk.

## 5 Conclusion

This study offers preliminary exploratory evidence that social anxiety, as an internalizing emotional indicator, is associated with IGD severity in a Saudi Arabian community sample, and that this association remains observable when an externalizing indicator (anger dyscontrol) is statistically controlled. Given the cross-sectional design and the limited psychometric properties of the screening tools employed, these findings should be understood as hypothesis-generating rather than as confirmatory evidence for distinct psychological pathways. The study is consistent with theoretical accounts emphasizing avoidance-based coping and online social preference as correlates of IGD, and highlights the value of examining both internalizing and externalizing emotional indicators simultaneously in future research using validated measures and longitudinal designs. As part of a broader program of research on IGD in Arabic-speaking contexts, this work contributes to a growing evidence base documenting IGD as a significant behavioral health concern across the Arab region (65–67) and underscores the importance of culturally informed approaches to understanding gaming-related problems among youth and young adults in Saudi Arabia and neighboring countries.

## Data Availability

The raw data supporting the conclusions of this article will be made available by the authors, without undue reservation.
